# 343. Relevance of Use of Anaerobic Blood Culture Bottle for the Diagnosis of Bacteremia

**DOI:** 10.1093/ofid/ofac492.421

**Published:** 2022-12-15

**Authors:** Taro Noguchi, Koh Shinohara, Yasuhiro Tsuchido, Satomi Yukawa, Masaki Yamamoto, Yasufumi Matsumura, Miki Nagao

**Affiliations:** Kyoto University Graduate School of Medicine, Kyoto, Kyoto, Japan; Kyoto University Graduate School of Medicine, Kyoto, Kyoto, Japan; Kyoto University Graduate School of Medicine, Kyoto, Kyoto, Japan; Kyoto University Graduate School of Medicine, Kyoto, Kyoto, Japan; Kyoto University Graduate School of Medicine, Kyoto, Kyoto, Japan; Kyoto University Graduate School of Medicine, Kyoto, Kyoto, Japan; Kyoto University Graduate School of Medicine, Kyoto, Kyoto, Japan

## Abstract

**Background:**

Blood cultures remain the cornerstone for the diagnosis of bacteremia. Although an anaerobic blood culture bottle can recover facultative and obligate anaerobes, routine use of an anaerobic bottle paired with an aerobic bottle remains controversial. We aimed to evaluate the role of anaerobic bottle for the diagnosis of bacteremia.

**Methods:**

We conducted a retrospective cohort study in a tertial hospital in Japan from January 2019 to September 2021. Blood culture results were collected from microbiology laboratory records. Blood cultures for which simultaneous pairs of aerobic and anaerobic bottles were collected and from which a single organism was recovered were included. Positive blood cultures were considered as a single episode of bacteremia when obtained within 14 consecutive days.

**Results:**

A total of 22677 blood cultures were collected, with 22650 bottles were paired. In 2962 paired blood cultures of which one or both bottles were positive, 1079 episodes of clinically significant monomicrobial bacteremia were observed. In these episodes, 898 had a positive aerobic bottle, while 789 had a positive anaerobic bottle, with 608 had both positive bottles. In 181 episodes (16.8%), clinically significant organisms were isolated in an anaerobic bottle without concomitant positive aerobic bottle. In these anaerobic bottles, 146 facultative and 35 obligate anaerobes were recovered. The most common facultative anaerobe was *Escherichia coli* (30 isolates), followed by *Staphylococcus aureus* (27 isolates). *Bacteroides fragilis* (11 isolates) was the most common obligate anaerobe.

**Conclusion:**

Recovery of clinically significant facultative and obligate anaerobes from anaerobic bottle alone was observed in 17% of episodes, which supports that the routine collection of both aerobic and anaerobic bottles is relevant.

**Disclosures:**

**All Authors**: No reported disclosures.

